# Pharmacological Evaluation of Total Alkaloids from *Nux Vomica*: Effect of Reducing Strychnine Contents

**DOI:** 10.3390/molecules19044395

**Published:** 2014-04-10

**Authors:** Jun Chen, Yange Qu, Dongyue Wang, Pei Peng, Hao Cai, Ying Gao, Zhipeng Chen, Baochang Cai

**Affiliations:** Engineering Center of State Ministry of Education for Standardization of Chinese Medicine Processing, Nanjing University of Chinese Medicine, Nanjing 210023, China

**Keywords:** *Strychnos nux-vomica* L., total alkaloids, antitumor, analgesic, anti-inflammatory, oral administration, pharmacokinetics, acute toxicity

## Abstract

The aim of the study was to investigate the possibility of improving the therapeutic efficacy of the total alkaloid fraction (TAF) extracted from processed nux vomica by reducing the strychnine contents. Most strychnine was removed from TAF to obtain the modified total alkaloid fraction (MTAF). The toxicity and pharmacokinetics of TAF and MTAF were further investigated and compared besides their antitumor, analgesic and anti-inflammatory activities. The results showed that the ratios of brucine to strychnine were 1:2.05 and 2.2:1 for TAF and MTAF, respectively, and the toxicity of TAF was about 3.17-fold higher than that of MTAF. Compared to brucine alone, the elimination of brucine was found to be inhibited by other alkaloids in TAF or MTAF except strychnine. Significantly increased pharmacological activities when administered by the oral route were obtained with MTAF in comparison to TAF and nux vomica powder (NVP). In summary, MTAF might replace NVP and TAF in the clinical application of Chinese medicine to obtain much higher efficacy.

## 1. Introduction

*Strychnos nux-vomica* L. (Loganiaceae) is a deciduous tree that grows in tropical areas and is distributed throughout India and Southeast Asia. The dried seed of this plant, nux vomica, has been applied clinically in Chinese medicine for hundreds of years. As a major ingredient, nux vomica has been frequently used in many proprietary Chinese medicines such as “Maqianzi Powder”, “Jiufen Powder”, “Fengshimaqian Tablet”, “Shufengdingtong Pill”, “Shenjinhuoluo Pill”, “Biqi Capsule”, *etc.*

**Figure 1:**
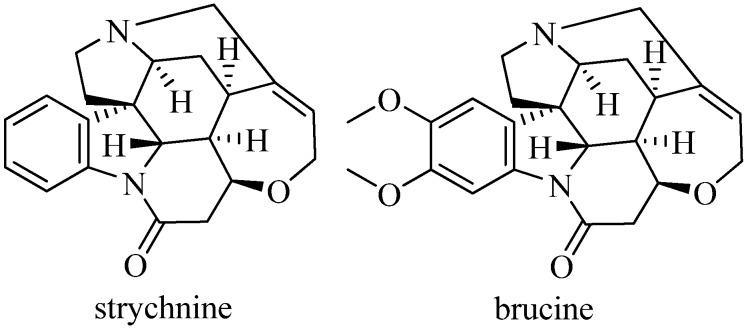
Chemical structures of strychnine and brucine.

The main bioactive constituents of nuxvomica are known to be alkaloids [1], responsible for both the pharmacological and toxic properties possessed by the seed. In our previous study, a total of 16 alkaloids have been separated from nux vomica and identified, among which strychnine and brucine (Figure 1) accounted for more than 50% of the total [2]. The toxic properties of these alkaloids, especially strychnine, have limited the clinical use and the investigation of nux vomica as a medicine. Therefore, in traditional oriental medicine, nux vomica needs to be properly processed so as to reduce its toxicity before it can be used in medicinal prescriptions. It was found that the content of strychnine was dramatically reduced by about 90% after processing by roasting in sea sands and boiling in water in comparison to the crude *Styrychno nux-vomica* seeds [3]. The significant decrease of strychnine content may account for the significantly decreased toxicity of the processed nux vomica.

Strychnine, the most abundant alkaloid of nux vomica, is highly toxic to humans and most domestic animals. Israeli authorities have prohibited its use, as have Britain and the European Union, yet it is still in use in some Asian countries [4]. Strychnine is a well-known potent antagonist of glycine receptors in the vertebrate central nervous system and a strong blocker of various types of muscle and neuronal nicotinic acetylcholine receptors [5,6]. Its poisoning is characterized by a short prodromal phase, after which there is an unusual combination of seizures with intact sensorium. Complications consist of hyperthermia, renal failure and rhabdomyolysis. The usual lethal dose of strychnine is reported to be between 50 and 100 mg, and the common cause of death is respiratory failure [7].

Strychnine had been proven to possess little therapeutic value with respect to antitumor, analgesic and anti-inflammatory activity. The analgesic activities of brucine and strychnine were compared by the acetic acid writhing test. The results showed that brucine had significant inhibition potency and the duration of its inhibition effect was about four times as long as that of pethidine. However, it was found that strychnine had no significant inhibition effect [8]. Brucine and brucine N-oxide have been proved to be mainly responsible for the analgesic effects produced by nux vomica [9]. In addition, strychnine was also found to possess less antitumor activity compared to brucine. After incubation with HepG2 liver cancer cells for 72 h, the IC_50_ values of strychnine and brucine were measured to be 0.52 and 0.10 mM respectively [10]. Brucine N-oxide was found to be ineffective on HepG2 cells proliferation [10].

It should be noted that in addition to its significantly enhanced therapeutic efficacy compared to strychnine, brucine is much less toxic. Following i.p. administration to mice, the LD_50_ values of strychnine and brucine were determined to be 1.10 and 50.10 mg/kg, respectively [11]. Therefore, formulations containing brucine have been intensively investigated [12,13,14].

Since strychnine possesses little therapeutic potency and high toxicity, it is highly possible that the removal of strychnine from nux vomica could result in significantly higher therapeutic efficacy. Therefore, in our previous study [15], the modified total alkaloid fraction (MTAF) was first obtained from the total alkaloid fraction (TAF) by removing most of the strychnine, which was extracted from crude nux vomica. Then the analgesic and anti-inflammatory activities of TAF, MTAF, brucine and strychnine dissolved in hydrogel were compared after transdermal administration. MTAF showed significant analgesic activity in all the chemical-, thermal- and physical-induced nociception models, which indicated the presence of both centrally and peripherally mediated activities besides significant anti-inflammatory activity against xylene-induced ear edema. However, TAF and strychnine demonstrated little activity in all those pharmacological tests.

The oral route is the most commonly used administration route for traditional Chinese medicines. and most prescriptions containing nux vomica are administered by the oral route. However, unlike the case of transdermal administration, administration of the raw seeds by the oral route is strictly forbidden and it must be processed before clinical use. Parching in a sand bath is the official standard method of processing nux vomica described and recorded in the Chinese Pharmacopoeia.

Strychnine represents up about 30%–50% in TAF composition extracted from the processed nux vomica. It is obvious that with the removal of most of the strychnine MTAF could display significantly increased therapeutic efficacy in antitumor, analgesic and anti-inflammatory applications. If MTAF can be applied in the clinical practice of Chinese medicine instead of TAF, it is reasonable to believe that the pharmacological effects of antitumor, analgesic and anti-inflammatory prescriptions containing nux vomica might be dramatically improved.

## 2. Results and Discussion

### 2.1. Alkaloid Composition of TAF and MTAF

**Figure 2:**
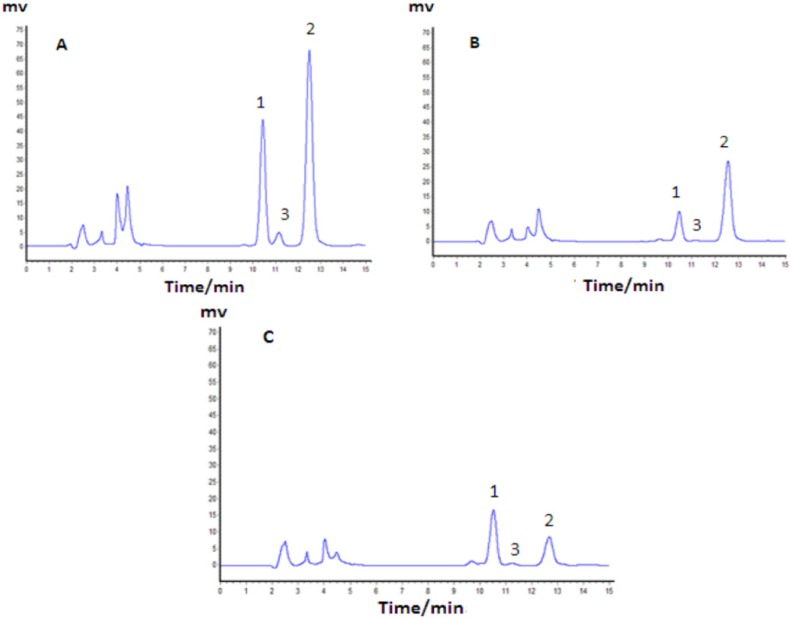
HPLC chromatographs of standard solution (**A**), TAF (**B**) and MTAF (**C**). Peaks: 1. Brucine; 2. Strychnine; 3. Brucine N-oxide.

The composition of TAF and MTAF were determined by HPLC analysis. The results showed that strychnine, brucine, and brucine N-oxide accounted for 42.35 ± 2.22%, 20.56 ± 0.95%, and 0.091 ± 0.007% in TAF (*n* = 5), respectively, while the corresponding percentages changed to 18.05 ± 1.07%, 40.67 ± 1.15%, and 0.250 ± 0.029%, respectively, in MTAF (*n* = 5). It was obvious that most of the strychnine was removed from MTAF and the ratio of brucine to strychnine changed from 1:2.05 to 2.2:1. The percentage of other alkaloids except strychnine and brucine were 38.09% and 41.29% in TAF and MTAF, respectively. HPLC chromatographs of TAF and MTAF are shown in Figure 2. In addition, compared to TAF, the yield of brucine in MTAF was calculated to be 92.69%.

Although brucine N-oxide possesses a higher therapeutic index compared to brucine for analgesic applications [9], it was not considered to be very important when TAF or MTAF was applied due to its relatively low content.

If extraction of TAF with 50% ethanol works so well to eliminate strychnine while still retaining brucine, perhaps it would be better to skip the initial processing with sand and boiling and simply prepare TAF, followed by further extraction with 50% ethanol.

### 2.2. Acute Toxicity Test

Table 1 shows a comparison of the acute toxicity of TAF and MTAF. The oral LD_50_ of MTAF was 15.10 mg/kg, while the LD_50_ value of TAF was 4.76 mg/kg. The toxicity of MTAF with the removal of most of the strychnine seemed to be significantly reduced compared with that of TAF. The acute toxicity test results revealed that the toxicity of TAF was about 3.17-fold higher than that of MTAF due to its much higher strychnine content. In all instances, the animals died of tonic seizures preceded by clonic convulsions.

**Table 1:** Toxic values of orally administrated TAF and MTAF in mice.

Formulations	LD_95_ (mg/kg)	LD_50_ (mg/kg)	LD_5_ (mg/kg)
TAF	10.47 (7.20–15.21)	4.76 (3.92–5.78)	2.17 (1.48–3.17)
MTAF	35.13 (23.26–53.05)	15.10 (12.35–18.47)	6.49 (4.29–9.84)

*Note*: 95% confidence interval in the brackets.

It should be noticed that strychnine might be much more toxic than brucine via the oral route because of its good absorption. In the processed nux vomica, the ratio of strychnine to brucine was about 2:1. However, after oral administration of the processed nux vomica, the mean maximum plasma concentrations of strychnine and brucine detected in the rats were 54.93 and 4.95 ng/mL, respectively [16]. The LD_50_ of brucine following oral administration to mice was determined to be 78 mg/kg [17], while the corresponding value of strychnine measured in our laboratory was 6.62 mg/kg. It is evident that strychnine is much more toxic than brucine.

### 2.3. Method Validation for HPLC Analysis of Brucine in Plasma

The selectivity of the method was evaluated by analyzing blank plasma samples prior to administration. The detection of huperzine A (IS) and brucine by HPLC was highly selective with no interference from other compounds after sample preparation. For the establishment of the calibration curve, seven concentrations of the analyte solution were prepared. The linear relationship (Y = 0.000899C + 0.021815, r = 0.9972) was kept over the concentration ranges from 50 to 2,000 ng/mL. The lower limit of quantification (LOQ) for brucine was 50 ng/mL. The RSDs of intra- and inter-day precisions of brucine were all within 10.0%. The extraction solvent used in the experiment showed good extraction efficiency. The relative recoveries of brucine from mouse plasma were in the range of 92.45%–97.66% at three QC levels (50, 300, 2000 ng/mL). Mean absolute recoveries of brucine from mouse plasma were in the range of 91.12%–93.78% at three QC levels.

The stability experiments aimed at testing samples under all possible conditions the samples might experience. The analyte was found to be stable in mouse plasma (RE within ± 15%) after three cycles of freezing (−20 °C) and thawing (room temperature). The analyte was also shown to be stable in the reconstituted solution for 12 h at room temperature (RE within ± 15%). No signs of degradation were found under the freeze condition (−20 °C) for 7 days. The results of the studies demonstrated that there were no significant degradations of brucine in plasma occurred under different experimental conditions.

### 2.4. Pharmacokinetics

**Figure 3:**
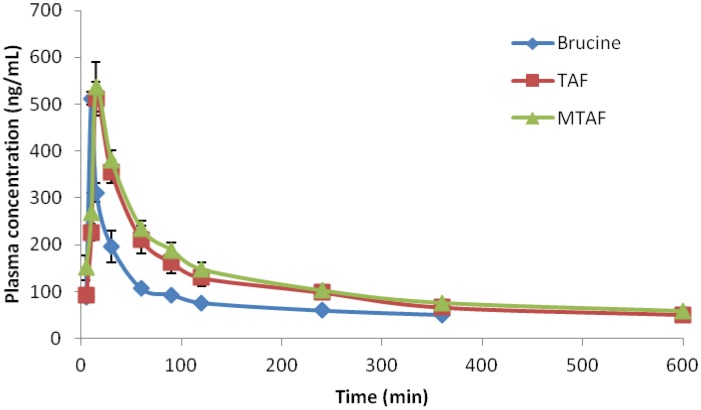
Plasma concentration *versus* time profile of brucine after oral administration of brucine, TAF and MTAF at the brucine dose of 1 mg/kg to rats (*n* = 6).

The mean plasma concentration-time curve profiles are illustrated in Figure 3. The pharmacokinetic parameters fitted a two-compartment model and are shown in Table 2. It was obvious that the AUC values of the TAF and MTAF groups were significantly higher than those of the brucine group. The AUC_0–__t_ values of TAF and MTAF groups were 1.69 and 1.89 times higher, respectively, than the corresponding value of the brucine group.

In addition, V and CL of brucine after administration of TAF and MTAF were markedly decreased. However, it was found that C_max_ was almost the same among brucine, TAF and MTAF groups. After oral administration, it appeared that the absorption of brucine was not affected by other alkaloid components in TAF or MTAF, but the elimination of brucine was found to be inhibited by other alkaloid components in TAF or MTAF.

**Table 2:** Pharmacokinetic parameters of brucine after oral administration of brucine, TAF and MTAF at the brucine dose of 1 mg/kg to rats (*n* = 6).

Parameters	Brucine	TAF	MTAF
AUC_0–__t_ (ng/mL·h)	555.30 ± 25.48	1092.43 ± 90.12 ***	1210.44 ± 66.71 ***^,△^
AUC_0–∞_ (ng/mL·h)	766.75 ± 21.77	1294.53 ± 111.40 ***	1449.95 ± 109.11 ***^,^^△^
MRT_0–∞_ (h)	4.60 ± 0.23	5.31 ± 1.20	5.46 ± 0.61 **
T_1/2_ (h)	3.61 ± 0.13	3.95 ± 0.91	4.22 ± 0.52 *
CL/F (L/h/kg)	1.32 ± 0.06	0.78 ± 0.06 ***	0.66 ± 0.06 ***^,^^△^
V/F (L/kg)	6.80 ± 0.36	4.39 ± 0.85 ***	4.20 ± 0.41 ***
T_max_ (h)	0.17	0.25	0.25
C_max_ (ng/mL)	512.72 ± 13.34	511.82 ± 36.65	537.07 ± 53.73

*Notes*: * *p* < 0.05, ** *p* < 0.01, *** *p* < 0.001 *vs.* the brucine group, ^△ ^*p* < 0.05 *vs.* the TAF group.

Following oral administration of TAF and nux vomica powder (NVP) at the same brucine dose, the AUC_0–∞_ values were determined to be 784 and 432 ng/mL h, respectively [18]. It seemed that alkaloids were absorbed completely when administration in the form of TAF. Therefore, the relatively higher pharmacological activity of TAF and MTAF compared with NVP might be partly due to the enhanced oral absorption of active alkaloids such as brucine. 

Besides strychnine and brucine, 14 alkaloids are also found in nux vomica, namely novacine, icajine, β-colubrine, isostrychnine, pseudostrychnine, pseudobrucine, 16-hydroxy-α-colubrine, vomicine, isobrucine, isobrucine N-oxide, isostrychnine N-oxide, 2-hydroxy-3-methoxystrychnine, brucine N-oxide, and strychnine N-oxide [2]. These alkaloids belong to the indole alkaloids class and have similar chemical structures. Therefore, it is possible that these alkaloids have similar metabolism reactions and thus inhibit the metabolism of brucine to increase its plasma concentration. 

### 2.5. Growth Inhibition Effects on Cancer Cells in vitro

The MTT assay was used as an indirect measure to determine the viability of cancer cells exposed to TAF or MTAF. It was found that both TAF and MTAF caused cell death in a concentration-dependent manner. The IC_50_ values were calculated and compared. As shown in Table 3, MTAF had much smaller IC_50_ values as compared to TAF, suggesting that the former was more effective against cancer cell proliferation than the latter. Furthermore, the anticancer activity was also dependent of cell type.

**Table 3:** IC_50_ values (μg/mL) of TAF or MTAF on the cancer cell growth after 72 h treatment.

Cancer cell lines	TAF	MTAF	Cisplatin
HepG2	70.71	38.58	<1
MGC-803	77.62	40.20	<1
A549	80.02	76.52	<1
A2780	32.79	15.67	1.45
LoVo	14.89	6.57	<1

### 2.6. Acetic Acid-Induced Writhing Response

Results obtained from acetic acid-induced writhing test in mice are presented in Table 4. Negative group and positive group were treated with normal saline and aspirin, respectively. NVP at the dose of 25 mg/kg, TAF at the dose of 1.0 mg/kg and MTAF at doses of 0.5 and 1.0 mg/kg all produced significant reduction in the number of abdominal writhing response induced by acetic acid with around 21.20%, 23.80%, 24.42%, and 34.78% of inhibition, respectively.

**Table 4:** Results of the acetic acid-induced writhing test after oral administration of different formulations to mice (*n* = 10).

Experimental groups	Dose (mg/kg)	Writhing times	PP (%)
Negative	-	36.89 ± 2.95	-
Aspirin	15	21.59 ± 3.78 **	41.47
NVP	25	29.07 ± 3.49 *	21.20
TAF	1.0	28.11 ± 3.28 **	23.80
MTAF	1.0	24.06 ± 2.72 ***	34.78
MTAF	0.5	27.88 ± 2.54 **	24.42
MTAF	0.25	33.07 ± 3.53	10.36

* *p* < 0.05, ** *p* < 0.01, *** *p* < 0.001 *vs.* the negative group.

### 2.7. Xylene-induced Ear Edema

The anti-inflammatory responses of different formulations on xylene-induced mouse ear edema are presented in Table 5. Statistical analysis demonstrated that NVP, TAF, MTAF and positive drug showed significant anti-inflammatory effects compared to negative group treated with normal saline. MTAF at 0.25 mg/kg was not shown significant activity, and the PIE of MTAF was 1.8-fold higher than that of TAF at the dosage of 1.0 mg/kg.

**Table 5:** Results of anti-inflammatory activity of different formulations on xylene-induced ear edema of mice (*n* = 10).

Experimental groups	Dose (mg/kg)	Extent of edema (mg)	PIE (%)
Negative	-	7.73 ± 1.34	—
Dexamethasone	15	3.23 ± 1.27 **	58.21
NVP	25	5.58 ± 2.67 *	27.81
TAF	1.0	5.96 ± 1.83 *	22.90
MTAF	1.0	4.52 ± 1.87 **	41.53
MTAF	0.5	5.09 ± 1.99 **	34.15
MTAF	0.25	6.32 ± 2.18	18.24

* *p* < 0.05, ** *p* < 0.01,*** *p* < 0.001 *vs.* the negative group.

### 2.8. Acetic Acid-Induced Vascular Permeability

As shown in Table 6, both TAF and MTAF showed reduced degrees of peritoneal inflammation produced by acetic acid in mice. The amount of dye leakage was dramatically decreased at TAF (1.0 mg/kg) and MTAF (0.5 and 1.0 mg/kg), while the effect of indomethacin (50 mg/kg) was the strongest. However, little activity was obtained with NVP.

**Table 6:** Effects of different formulations on acetic acid-induced vascular permeability in mice (*n* = 10).

Experimental groups	Dose (mg/kg)	Amount of dye leakage (μg)	Inhibition (%)
Negative	-	72.05 ± 7.00	-
Indomethacin	50	51.67 ± 9.58 ***	28.29
NVP	25	63.44 ± 10.31	11.95
TAF	1.0	59.58 ± 8.69 **	17.31
MTAF	1.0	55.05 ± 10.33 **	23.59
MTAF	0.5	59.49 ± 18.99 *	17.43
MTAF	0.25	67.10 ± 14.55	6.87

Compared to the negative group, * *p* < 0.05, ** *p* < 0.01.

The results of pharmacological evaluation revealed that the therapeutic efficacies were significantly increased by the usage of MTAF instead of NVP. Although the dose of MTAF was only one fortieth of NVP, the analgesic and anti-inflammatory activities were still dramatically higher than the latter. In addition, compared with TAF at the same dose or concentration, the antitumor, analgesic and anti-inflammatory activities of MTAF were also found to be higher than TAF. Although the significantly reduced contents of strychnine might account for the increased therapeutic efficacy and reduced toxicity of MTAF, it should be noted that the modified ratio of strychnine to brucine might be also relevant.

## 3. Experimental

### 3.1. Collection and Identification

The seeds of *Strychnos nux-vomica* L., nux vomica, were collected from Yunnan Province, China and identified by Professor Baochang Cai (Nanjing University of Chinese Medicine, Nanjing, China) according to their morphological characteristics. The voucher specimens were deposited in the herbarium of Engineering Center of State Ministry of Education for Standardization of Chinese Medicine Processing.

### 3.2. Processing of Nux Vomica and Preparation of NVP

According to the Chinese Pharmacopoeia, sand in an iron pan was first heated up to 220–230 °C (the temperature at the bottom of the sand). Then nux vomica was put into the heated sand and parched for 3 min. The seeds, nux vomica, became dark yellow in color and were well swollen. The processed nux vomica was ground into a fine powder and passed through a sieve (50 mesh) to obtain NVP.

### 3.3. Extraction of TAF from the Processed Nux Vomica

The powder (100 g) was extracted three times with 70% ethanol (1.5 L) under reflux for 0.5 h each time. After filtration, the filtrates were combined and concentrated by rotary evaporation under reduced pressure. Then, the residue was dissolved in 1 mol/L hydrochloric acid (200 mL) and separated by centrifugation at 4,000 rpm for 10 min. The pH of the supernatant was adjusted to 12.0 with 40% sodium hydroxide. The solution was extracted six times with dichloromethane (100 mL each) and the extraction was evaporated to dryness under vacuum. Finally, 2.60 g TAF was obtained (yield: 2.60%, w/w). 

### 3.4. Preparation of MTAF

The solubility of brucine in 50% ethanol was 455.40 mg/mL, while that of strychnine was only 1.60 mg/mL. Therefore, by using 50% ethanol, most strychnine was precipitated while most brucine and other alkaloid components remained dissolved in the solution. The TAF powder (2.60 g) was dissolved in 50% ethanol (1.3 L), and was concentrated to 100 mL under reduced pressure. The concentrated solution was stored in the refrigerator at 4 °C overnight and then was separated by centrifugation at 4,000 rpm for 10 min. The supernatant was evaporated to dryness under vacuum and 0.808 g MTAF was obtained (yield: 31.08%, w/w).

### 3.5. Experimental Animals

Pharmacological experiments except hot plate test were performed with ICR mice of both sex, weighing 18–22 g. Pharmacokinetic experiments were carried out with SD rats of both sex. Both mice and rats were obtained from the Experimental Animal Center of Nanjing Medical University (Nanjing, China). They were housed in plexiglass cages at 22 ± 2 °C, relative humidity 55 ± 5% with 12 h light/12 h dark cycle and provided with standard pellet diet with tap water *ad libitum*. Animal experiments were performed in accordance to the Principles of Laboratory Animal Care and Use in Research (Ministry of Health, Beijing, China). The protocols of animal experiments were approved by the Animals Ethics Committee of Nanjing University of Chinese Medicine. Animals were acclimatized to the laboratory environment for at least 2 h before testing, were used only once during the experimental protocol.

### 3.6. Drugs, Chemicals, and Chemical Reagents

Strychnine, brucine, brucine N-oxide, Hoechst 33258, and 3-(4,5-dimethythiazol-2-yl)-2,5- diphenyltetrazolium bromide (MTT) were supplied by Sigma-Aldrich Chemical Co. (St. Louis, MO, USA). The internal standard (IS) huperzine A was supplied by National Institute for the Control of Pharmaceutical and Biological Products (Beijing, China). HPLC grade acetonitrile was purchased from Tedia (Fairfield, OH, USA). Distilled water, prepared from demineralized water, was used throughout the experiments. All the other chemicals and chemical reagents were of analytical grade and purchased from Nanjing Chemical Reagent Corporation (Nanjing, China).

### 3.7. HPLC Analysis of Strychnine, Brucine, and Brucine N-oxide in TAF and MTAF

A Shimadzu HPLC system (Kyoto, Japan) consisting of a LC-20AT pump, a SPD-20A UV-VIS detector was used for the simultaneous assay of strychnine and brucine in TAF or MTAF. The mobile phase consisted of acetonitrile and buffer (10 mM sodium heptane sulfonate and 20 mM potassium dihydrogen phosphate, adjusted pH to 2.8 with 10% phosphonic acid) , which was selected according to the Chinese Pharmacopoeia with minor modification. The ratio of acetontrile:buffer (v/v) was adjusted to 24:76. Separation was carried out at 35 °C using a Superstar reverse-phase C_18_ column (250 mm × 4.6 mm, 5 μm, Hanbang Corp., China). The detection wavelength was set at 264 nm and a flow rate of 1.0 mL/min was employed. A sample volume of 10 μL was injected. TAF or MTAF were dissolved in dichloromethane to obtain 1 mg/mL solution. Then the TAF or MTAF solution was further diluted with methanol to 10 μg/mL and injected into HPLC. The contents of strychnine and brucine were determined compared to a standard curve.

### 3.8. Acute Toxicity Test

TAF or MTAF was dissolved in normal saline and administered by oral route. The maximal and minimal lethal doses were preliminarily estimated. The dosages for determination of LD_50_ were then calculated according to the ratio of dose for each group and administered to five dosage groups (10 mice each). All mice were observed for general symptoms. The number of dead mice was recorded. LD_95_, LD_50_, and LD_5_ values were calculated using the Bliss method [19].

### 3.9. Pharmacokinetic Study

Apparatus and chromatographic conditions were the same as described in Section 3.7. Plasma samples were treated using liquid-liquid extraction step described previously [20]. Briefly, plasma (100 μL) was spiked with IS (10 μL, 40 μg/mL huperzine A solution in methanol). Aqueous ammonia (10 μL) was added and the samples were made alkaline. *n*-Hexane-dichloromethane-isopropanol (3 mL, 65:30:5, v/v/v) was then added. The mixture was vortex-mixed for 3 min and subsequently was centrifuged for 10 min at 4,000 rpm at ambient temperature. The organic layer was transferred into a glass tube and the residue was re-extracted with chloroform in a similar manner. The combined organic layer was concentrated at 50 °C under a gentle stream of nitrogen gas, until a completely dried residue was left. The residue was reconstituted in methanol (100 μL) and centrifuged for 5 min at 12,000 rpm. Finally, a portion of the supernatant (50 μL) was injected into the HPLC system for analysis. Method validation was performed as our previous paper [20]. Linearity of the calibration curve, accuracy, precision, extraction recovery and stability of the plasma samples were investigated.

Eighteen rats were divided randomly into three groups. The tested animals were orally administered with brucine, TAF and MTAF solution at the brucine dose of 1 mg/kg, respectively. At predetermined intervals, the rats were anaesthetized using ether and blood samples were collected from the retro-orbital plexus into heparinized microfuge tubes. The blood samples were then immediately centrifuged at 10,000 rpm for 2 min to obtain the plasma samples, which were then stored at −20 °C before HPLC analysis.

The following pharmacokinetic parameters were calculated using non-compartmental analysis of data (Drug and Statistics ver. 2.0 Program, Shanghai, China): the area under the plasma concentration-time curve from time zero to time infinity (AUC), the total body clearance (CL), the mean residence time (MRT), the terminal elimination half-life (T_1/2_), and the apparent volume of distribution at steady state (V_ss_). The observed peak plasma concentration (C_max_) and the corresponding time (T_max_) were directly obtained from the raw data.

### 3.10. Growth Inhibition Effects on Tumor Cells in Vitro

Human hepatoma HepG2 cancer cells, human gastric cancer MGC-803 cells, human lung cancer A549 cells, human colon cancer LoVo cells, and human ovarian cancer A2780 cells were purchased from Kaiji Biotechnology Inc. (Nanjing, China). Cells were cultured at 37 °C in a 5% CO_2_/95% air humidified atmosphere in RPMI 1640 medium containing 25 mM HEPES and 2 mM L-glutamine supplemented with 20% (v/v) heat-inactivated FCS, 100 IU/mL penicillin and 100 mg/mL streptomycin.

Tumor cells were seeded in 96-well plates at a density of 6 × 10^4^ cells/well (in triplicates). The cells were treated with TAF or MTAF at varying concentrations (12.5–200 μg/mL). Cell growth was assessed after 72 h by MTT assay and the optical densities were measured at 490 nm. Cells that did not receive any drug (control) were considered as having 100% cell growth and growth from treated cells was compared with this value (percent growth *versus* control). The growth inhibition effects of cisplatin on tumor cells were also evaluated as a positive control.

### 3.11. Acetic Acid-Induced Writhing Response

Abdominal constriction induced by the i.p. injection of acetic acid was carried out according to method described previously [10] with minor modifications. Negative and positive groups were orally treated with normal saline and the aspirin (15 mg/kg), respectively. Thirty minutes after the oral administration of different formulations, acetic acid (1%, 10 mL/kg) was injected intraperitoneally. Each animal was observed in an individual box within 25 min immediately after the i.p. injection of acetic acid. The number of writhing and stretching response was cumulatively counted and the percentage protection (PP) was calculated using the following expression:

PP (%) = ((negative mean − treated mean)/negative mean) × 100%
(1)

### 3.12. Xylene-Induced Ear Edema

An edema was induced on the right ear by topical application of xylene (50 μL) on the inner and outer surface. Half an hour later, different formulations (NVP, TAF and MTAF) were administered orally. Negative and positive groups were orally treated with normal saline and the dexamethasone (15 mg/kg), respectively. After 30 min, the animals were sacrificed by cervical dislocation. Disks of 6 mm diameter were removed from each ear and weighed in balance. The extent of edema expressed as the weight difference between the right and the left ear disks of the same animal. The anti-inflammatory activity was expressed as percentage of the inhibition of edema (PIE). PIE was calculated using the following formula:

PIE(%) = ((negative mean − treated mean)/negative mean) × 100%.
(2)

### 3.13. Acetic Acid-Induced Vascular Permeability

Mice were orally treated with different formulations. Negative and positive groups were orally treated with normal saline and the indomethacin (50 mg/kg), respectively. After 30 min, the mice were injected (i.p.) with 0.25 mL of 0.6% acetic acid solution. After another 30 min, 10 mL/kg of 10% (v/v) Evan’s Blue was injected intravenously through the tail vein and the mice were killed at 30 min after the injection. The viscera was exposed and irrigated with distilled water over a Petri dish. The elution was filtered and constituted to 10 mL, the dye leaking out into the peritoneal cavity was measured by spectrophotometry at 610 nm.

## 4. Conclusions

In summary, by removing most strychnine from TAF, MTAF with significantly reduced toxicity was obtained. After oral administration, the elimination of brucine might be inhibited by other alkaloids in TAF or MTAF and this resulted in increased bioavailability. Significantly increased analgesic and anti-inflammatory activities via the oral route were obtained with MTAF in spite of the fact that the dose of MTAF was reduced to only one fortieth that of NVP. In addition, compared with TAF, the pharmacological activities of MTAF were also found to be increased. It is suggested that MTAF can replace with NVP and TAF in the antitumor, analgesic and anti-inflammatory prescriptions in Traditional Chinese Medicine to obtain much higher therapeutic efficacy.
